# From Pixels to Pathology: Employing Computer Vision to Decode Chest Diseases in Medical Images

**DOI:** 10.7759/cureus.45587

**Published:** 2023-09-20

**Authors:** Muhammad Arslan, Ali Haider, Mohsin Khurshid, Syed Sami Ullah Abu Bakar, Rutva Jani, Fatima Masood, Tuba Tahir, Kyle Mitchell, Smruthi Panchagnula, Satpreet Mandair

**Affiliations:** 1 Department of Emergency Medicine, Royal Infirmary of Edinburgh, National Health Service (NHS) Lothian, Edinburgh, GBR; 2 Department of Allied Health Sciences, The University of Lahore, Gujrat Campus, Gujrat, PAK; 3 Department of Microbiology, Government College University Faisalabad, Faisalabad, PAK; 4 Department of Internal Medicine, Youjiang Medical University for Nationalities, Baise, CHN; 5 Department of Internal Medicine, C. U. Shah Medical College and Hospital, Gujarat, IND; 6 Department of Internal Medicine, Gulf Medical University, Ajman, ARE; 7 Department of Business Administration, Iqra University, Karachi, PAK; 8 Department of Internal Medicine, University of Science, Arts and Technology, Olveston, MSR; 9 Department of Internal Medicine, Ganni Subbalakshmi Lakshmi (GSL) Medical College, Hyderabad, IND; 10 Department of Internal Medicine, Medical University of the Americas, Charlestown, KNA

**Keywords:** machine learning, artificial intelligence, nuclear medicine, ultrasounds, ct scans, mri, lesion detection, computer vision, chest x-ray, medical imaging

## Abstract

Radiology has been a pioneer in the healthcare industry's digital transformation, incorporating digital imaging systems like picture archiving and communication system (PACS) and teleradiology over the past thirty years. This shift has reshaped radiology services, positioning the field at a crucial junction for potential evolution into an integrated diagnostic service through artificial intelligence and machine learning. These technologies offer advanced tools for radiology's transformation. The radiology community has advanced computer-aided diagnosis (CAD) tools using machine learning techniques, notably deep learning convolutional neural networks (CNNs), for medical image pattern recognition. However, the integration of CAD tools into clinical practice has been hindered by challenges in workflow integration, unclear business models, and limited clinical benefits, despite development dating back to the 1990s. This comprehensive review focuses on detecting chest-related diseases through techniques like chest X-rays (CXRs), magnetic resonance imaging (MRI), nuclear medicine, and computed tomography (CT) scans. It examines the utilization of computer-aided programs by researchers for disease detection, addressing key areas: the role of computer-aided programs in disease detection advancement, recent developments in MRI, CXR, radioactive tracers, and CT scans for chest disease identification, research gaps for more effective development, and the incorporation of machine learning programs into diagnostic tools.

## Introduction and background

Radiology stands out as a medical field that swiftly embraced digital technology. From the 1970s onwards, various techniques such as computed tomography, positron emission tomography, digital mammography, single photon emission computed tomography, magnetic resonance imaging, digital ultrasound, and computed radiography have progressively gained significance in radiology. Initially, film copies were employed for the scrutiny, distribution, and preservation of these digital images. However, the late 1990s marked the inception of a transition towards a film-free, digital paradigm within radiology operations [1,2]. This shift gained momentum as retrieval, display, and transmission methods evolved, ushering in digital information storage techniques. Notably, the picture archiving and communication system (PACS) has entirely supplanted conventional X-ray film usage [3]. The healthcare system at large now enjoys extensive access to X-ray images as a result of substantial investments in digital technology within the radiology domain [4]. The advent of digital radiography has paved the way for significant strides in image-guided surgery and radiation therapy. However, it wasn't until the emergence of teleradiology that radiology could extend its reach on a global scale [5,6]. Teleradiology has now become the standard of care in numerous countries, including the United States. To effectively brace themselves for the forthcoming technological disruption brought about by artificial intelligence and machine learning (ML), radiology services have amassed vast repositories of digital images, some of which are stored in cloud-based archives.

During the mid-1980s, researchers in the radiology field embarked on their exploration of computer-aided diagnosis (CAD) as a tool intended to assist radiologists [7]. Subsequently, machine learning methods have gained prominence, especially since the mid-2000s, and they have now established widespread utility in situations demanding data categorization and analysis. The realm of machine learning (ML) has witnessed a remarkable surge in related articles, escalating from a mere few thousand in the early 2000s to approximately 35,000 by 2018. Impressively, around 85% of these articles have centered on the application of neural networks [8]. Diverse domains, including geosciences, drug development, quantum chemistry, autonomous vehicles, computational biology, and astronomy, have harnessed the capabilities of neural networks. Within the radiology community, several CAD tools have been developed, showcasing notable levels of sensitivity and specificity. Nonetheless, despite these advancements, a significant portion of these concepts have not been translated into practical clinical implementation within the radiology domain. The advent of digital mammography and its proven efficacy has fueled optimism among medical practitioners regarding breast cancer screening. Many clinicians are enthusiastic about incorporating CAD technology into their clinics. Simultaneously, there exists a perspective that the introduction of these innovative technologies may potentially render radiographers obsolete. In 2017, under the guidance of the Interagency Working Group on Medical Imaging and the Committee on Science, the National Science and Technology Council formulated a strategic blueprint to steer the trajectory of future imaging research and development [9]. This document strives to anticipate transformations across four pivotal facets of medical imaging research. Examples encompass the promotion of imaging services, streamlined patient referrals to these services, workflow restructuring to amplify efficiency, and the propagation of best practices within medical imaging.

## Review

Service description: radiology operations

**Figure 1:**
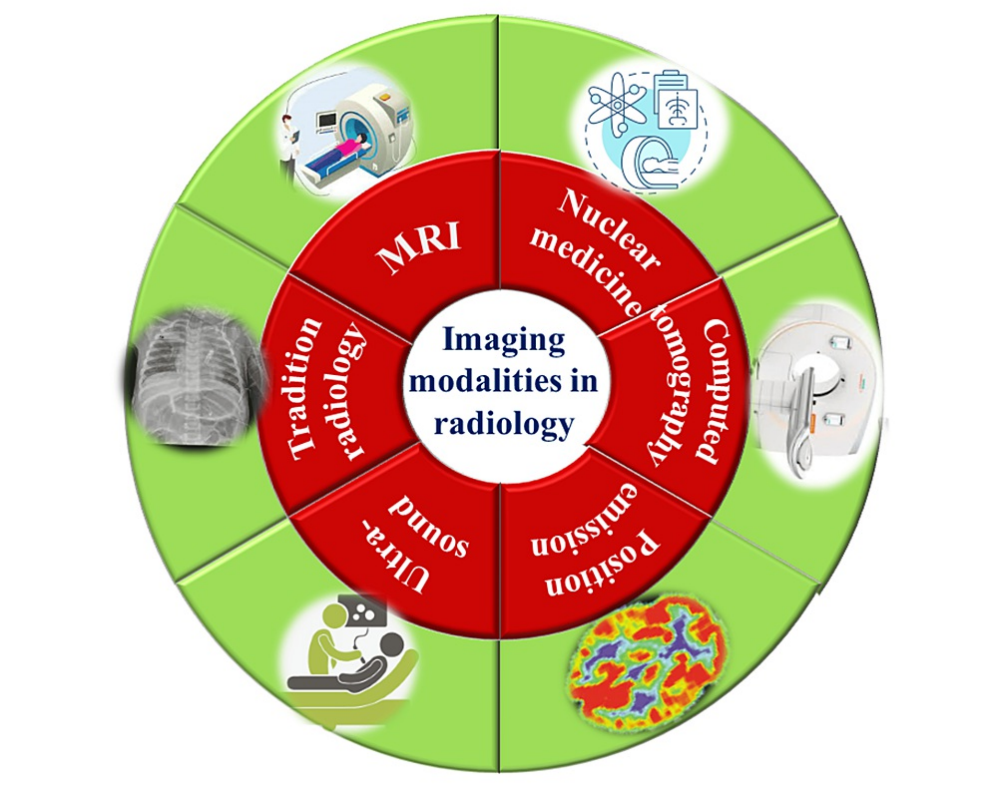
Radiology uses dozens of imaging modalities. Note: This image is the author's own creation.

The intricate nature of radiology services significantly contributes to their current low productivity levels. A substantial enhancement in overall productivity and efficiency is imperative. The radiology department operates within a complex framework, involving multiple tiers of staff, diverse equipment types, and time-critical information management. These factors collectively facilitate the delivery of therapeutic services to referring physicians and their patients. Each day, the average radiology department performs approximately fifty distinct types of imaging tests, spanning across various regions of the body. This diverse range of tests is facilitated by numerous imaging modalities, such as magnetic resonance imaging, nuclear medicine, computed tomography, positron emission tomography, ultrasound, and traditional radiography systems. A pivotal component in this operational landscape is the radiology information system (RIS) or picture archiving and communication system (PACS). These systems store and facilitate the sharing of digital images, efficiently managing the acquired images [10]. Referring physicians play a vital role in initiating imaging investigations, considering the patient's symptoms and medical history. In this intricate ecosystem, a radiologist, a medical specialist adept in utilizing imaging techniques for diagnosing and treating diseases, assumes a central role. Once an imaging study concludes within the imaging system, the PACS orchestrates the compilation of all images. It subsequently generates a tailored work list for each radiologist, aligning with the radiologist's specific area of expertise and conforming to the department's established protocols and procedures [4] (Figure 1).

As outlined in the study, a significant portion of a radiologist's time is dedicated to tasks such as analyzing and interpreting images, crafting reports, and engaging in consultations with referring physicians and patients [11]. The education of residents and fellows presents a substantial responsibility for academic departments. The duration required for comprehending various studies can notably vary based on the specific area under investigation. The research underscores that radiologists possess a notable proficiency in identifying anomalies within images [12]. In recent years, the time allocated by radiologists for image interpretation has shown a consistent upward trend. This trend corresponds to the evolving landscape of imaging technology, which has led to an increase in the volume of images acquired for each diagnostic investigation. For instance, spanning from 1999 to 2010, the average count of images obtained during a computed tomography (CT) scan surged from 82 to 679. Similarly, the count of magnetic resonance imaging scans performed witnessed an escalation from 164 in 2012 to 570 in 2015 [13]. These accumulated images can be compared over varying time periods to assess whether a patient's health has progressed or declined. However, this manual comparison process demands substantial effort from radiologists [14,15]. In most cases, picture archiving and communication system (PACS) and radiology information systems (RIS) utilize speech recognition technology to automatically generate reports once the radiologist completes the interpretation of a study. While there are rare instances where the information might stand alone, the generated report frequently serves as a point of reference for subsequent investigations [16]. A comprehensive understanding of the patient's condition necessitates the integration of various diagnostic tools, including radiological imaging, blood chemistry tests, and tissue samples. This amalgamation is crucial in forming a holistic view of the patient's health status.

Deep learning for CXR image-based chest disease detection

**Figure 2:**
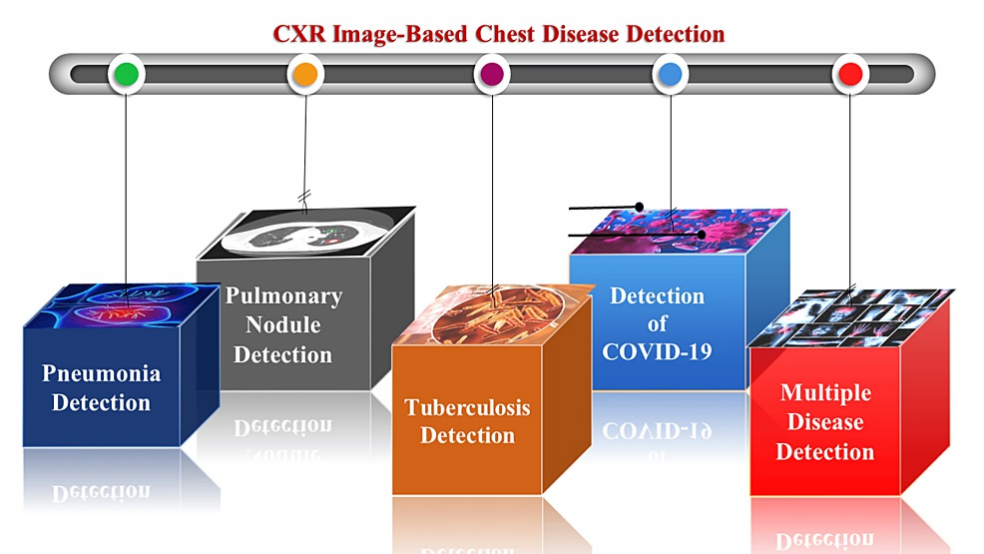
Deep learning for CXR image-based chest disease detection. CXR: chest X-ray. Note: This image is the author's own creation.

Several distinct computer-aided diagnosis (CAD) systems are available, each possessing its own distinct approach to identifying lung diseases. Timely diagnosis and intervention hold the potential to effectively manage chest diseases, particularly when detected at an early stage. The advanced stages of diseases, such as tuberculosis (TB), pneumonia, and COVID-19, often lead to more severe consequences. Chest X-ray (CXR) scans can exhibit one of three primary types of abnormalities. Firstly, there are abnormalities in texture, discernible through widespread alterations in the visual appearance and structure of the region. The second type, referred to as aberrant form anomalies, is characterized by deviations from the usual overall morphology. Lastly, focal abnormalities manifest as localized shifts in density within the image. This article undertakes a comprehensive survey of recently reported deep learning (DL) approaches, categorizing them based on the specific disease types they were developed for. The selection of diseases included in this survey is based on the mortality and prevalence rates provided by the World Health Organization (WHO) (Figure 2).

Pneumonia detection

Radiologists often encounter a complex challenge when attempting to identify pneumonia using CXR images. Pneumonia can exhibit similar characteristics to other illnesses or might even appear unremarkable in certain individuals [17]. For instance, a fully connected layer Swin transformer model has been employed to extract features from CXR images for pneumonia detection [18]. This model was trained using data from the pediatric-CXR and ChestX-ray8 datasets, and its performance was evaluated alongside that of deep convolutional neural network (DCNN) models. Through the implementation of image enhancement and data augmentation techniques, the provided model's accuracy increased from 87.30% on ChestX-ray8 to 97.20%. Another approach involves CXR image classification utilizing a deep convolutional neural network (CNN) model enriched with attention mechanisms, focusing on two classes: normal and pneumonia [19]. Training this model, ResNet50 with attention achieved an accuracy of 95.73% when using images from the pediatric-CXR dataset. Using CXR images from the Radiological Society of North America (RSNA)-pneumonia-CXR dataset, two deep convolutional neural network models (Inception-V4 and ResNet-50) were employed for the binary classification of pneumonia patients through transfer learning [20]. During validation, accuracy reached 94.00%, with Inception-V4 surpassing ResNet-50.

**Figure 3:**
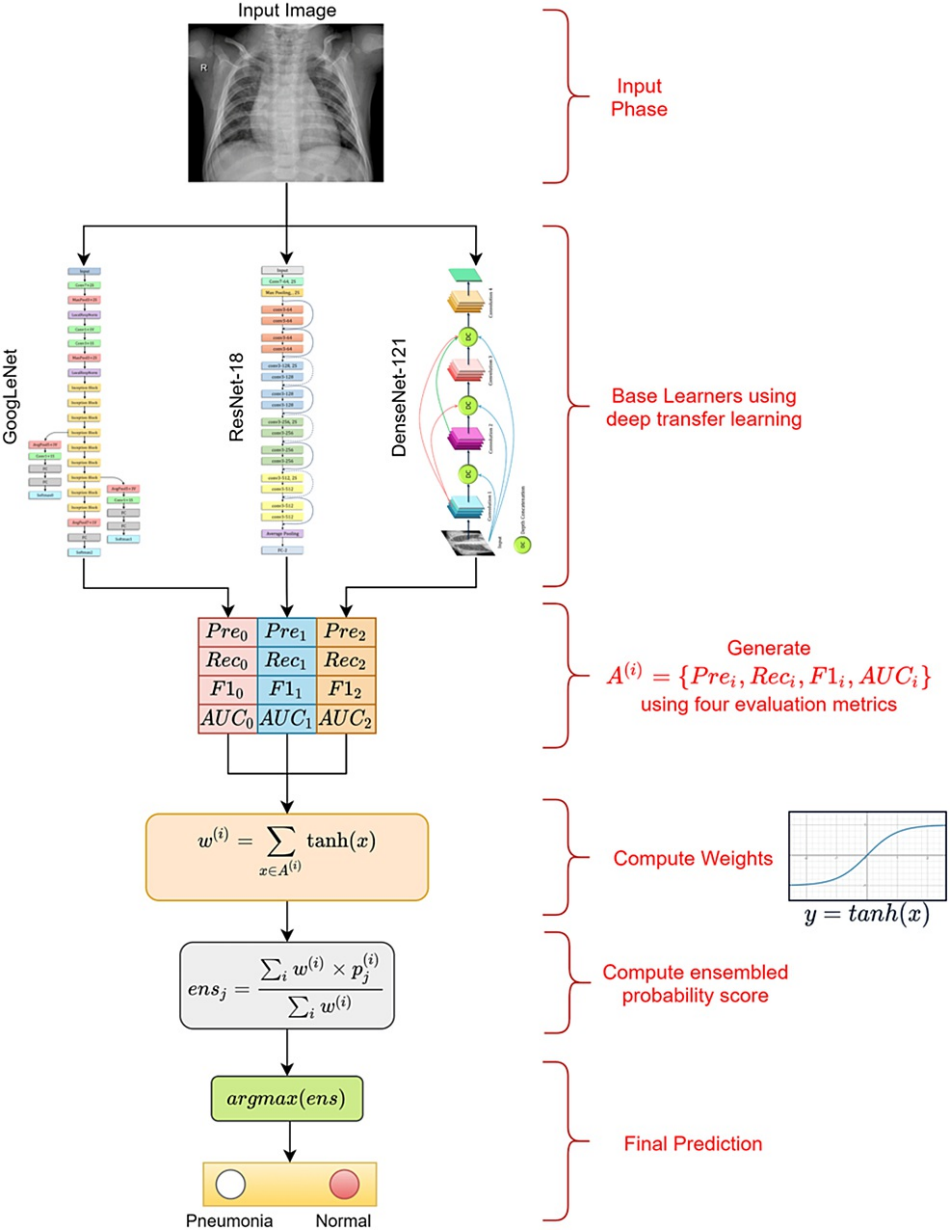
Proposed mechanism of the pneumonia detection framework. Reproduce under the terms of the Creative Commons Attribution License from Reference [22]. Copyright 2021 PLoS ONE.

In the context of pneumonia detection, the development of the 121-layer convolutional network CheXNet is notable [21]. This network is designed to detect and localize areas of the lungs affected by pneumonia. By fine-tuning the model, researchers replaced the last fully connected layer with a single-output layer after training on the ChestX-ray14 dataset. CheXNet achieved an area under the curve (AUC) of 76.80%, demonstrating its effectiveness. Another research avenue involves the use of transfer learning to create a computer-aided diagnosis (CAD) system for binary pneumonia categorization [22]. An ensemble approach using three separate DCNN models (GoogleNet, DenseNet-121, and ResNet-18) was employed alongside a five-cross-validation process. Utilizing the freely available pediatric-CXR and RSNA-pneumonia-CXR datasets, the proposed model achieved an accuracy of 98.81% on the pediatric-CXR dataset and 86.86% on the RSNA-pneumonia-CXR dataset (Figure 3).

In the pursuit of classifying images as either normal or indicating pneumonia, researchers leveraged binary classification techniques on a dataset of 5,856 chest X-ray (CXR) images sourced from the pediatric-CXR dataset [23]. Data augmentation techniques were employed as a means of expanding the dataset. Through these efforts, a custom convolutional neural network (CNN) was trained using the accumulated data to accurately detect instances of pneumonia, achieving an accuracy rate of 83.38%. Further advancements in research have aimed to distinguish between viral and non-viral pneumonia through confidence-aware anomaly detection [24]. This investigation heavily relied on the X-viral and X-COVID-19 CXR databases. The former contained 5,977 images showcasing viral pneumonia cases, while the latter encompassed 37,393 images illustrating non-viral pneumonia cases. Additionally, the second dataset comprised 107 images that were not infected with COVID-19 and 106 images that were infected. JF Healthcare's telemedicine software was deployed across 390 hospitals for both datasets. The proposed confidence-aware anomaly detection (CAAD) model exhibited robust performance, boasting a high area under the curve (AUC) value of 87.57%. This AUC value signifies the model's efficacy in identifying instances of viral pneumonia. The utilization of an 18-layer deep convolutional neural network (CNN) method facilitated the categorization of CXR images into pneumonia or normal categories [17]. This task was executed using the pediatric-CXR dataset. The resultant model achieved an impressive sensitivity (SEN) of 99.50%, accuracy (ACC) of 94.39%, and specificity (SPE) of 86.00%.

**Figure 4:**
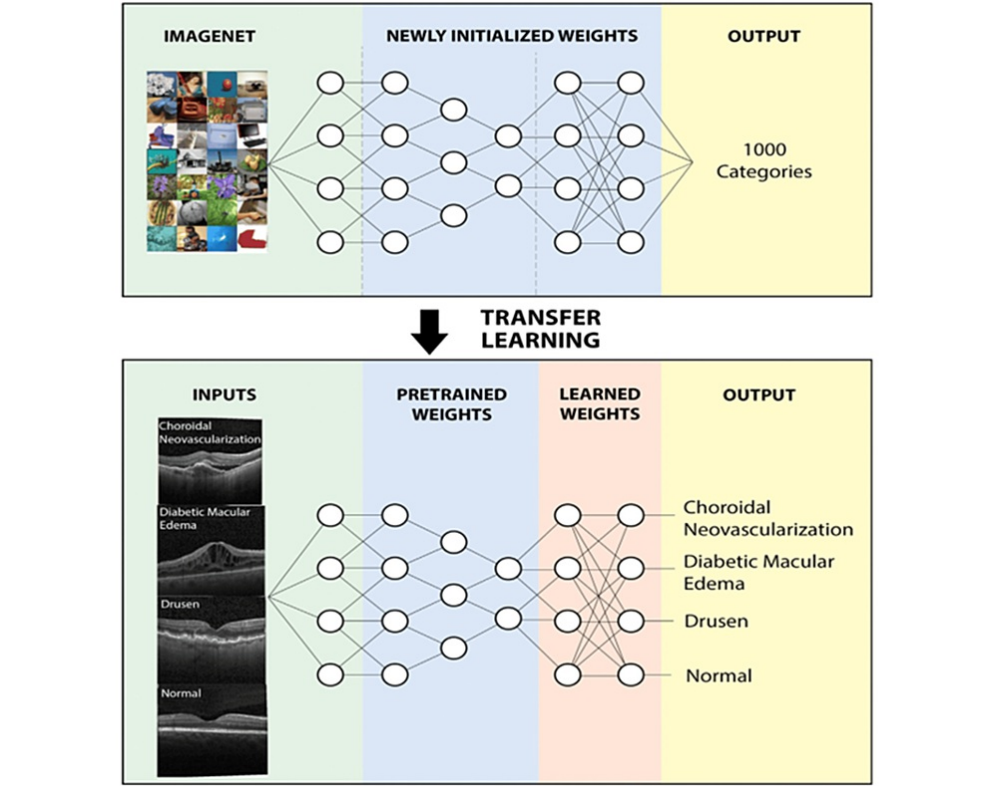
Mechanism of disease diagnosis by image-based deep learning. Reproduced with permission from Reference [26]. Copyright 2018 Elsevier.

In a similar vein, another study employed the pediatric-CXR dataset to train a deep convolutional neural network (DCNN) model for extracting features and diagnosing pneumonia [25]. Various data manipulation techniques, including resizing, flipping, and rotation, were applied to alter the dataset. The model was subjected to testing scenarios involving dropout and augmented data. The model's performance was notably enhanced through the integration of dropout mechanisms and augmented data, ultimately achieving an accuracy of 90.00%. In the realm of clinical decision support systems for medical imaging, trust and comprehension concerns prevail. This study introduces a deep learning-based diagnostic tool to detect potentially treatable forms of common retinal diseases that could lead to blindness. Through a transfer learning approach, this method requires significantly less data than is typically necessary to train neural networks. The application of this approach to an optical coherence tomography image database produced competitive findings with those derived from human experts in diagnosing diseases like age-related macular degeneration (AMD) and diabetic macular edema (DME). Additionally, the study showcases the AI system's potential by employing it for the diagnosis of juvenile pneumonia through chest X-ray images. This approach holds the promise of enhancing therapeutic outcomes by expediting the identification and referral of patients with treatable illnesses (Figure 4) [26].

An accuracy (ACC) of 90.70% was achieved through the utilization of the Inception-V3 model, along with a positive predictive value of 92.80% in identifying normal cases while assessing the likelihood of viral vs. bacterial pneumonia. Additionally, a new deep convolutional neural network (DCNN) model was proposed and trained using the pediatric-CXR dataset, with the aim of identifying and classifying instances of pneumonia [27]. To address overfitting and generalization errors caused by the dataset's limited size, several data-augmentation techniques were applied. Remarkably, the model exhibited a relatively high accuracy rate of 93.73%.

Pulmonary nodule detection

**Figure 5:**
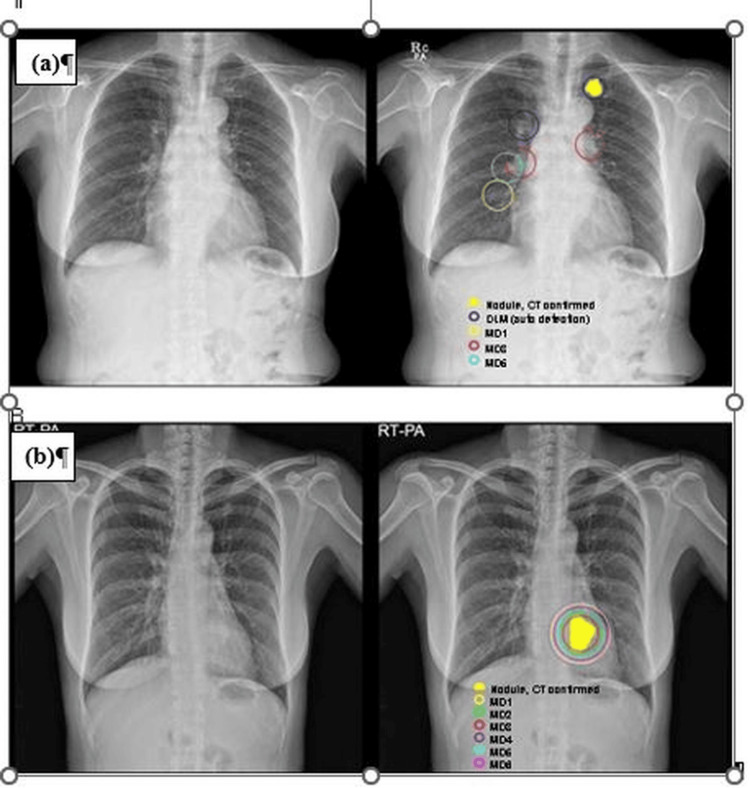
(a) Shows examples of lung cancer that were overlooked by DLM and by humans. A lung carcinoma of 1.8 cm in size was found by DLM in the left upper lobe but was hidden by the left first costal cartilage. (b) However, none of the human onlookers saw anything out of the ordinary. A lung carcinoma measuring 3 cm is seen in the retrocardiac region and the left lower lobe. Six human observers saw the lung mass, but DLM didn't see the abnormalities. DLM: deep learning model. Reproduced with permission from Reference [30]. Copyright 2019 Wolters Kluwer Health.

According to the World Health Organization, lung cancer has a high mortality rate, ranking as the leading cause of death among males and the third among females [28]. Lung nodules serve as indicators of lung cancer, and early detection through imaging technology is crucial for effective treatment and preventing metastasis and further complications. Multiple studies have highlighted the potential of deep learning (DL)-based systems to assist radiologists in detecting lung nodules within diagnostic images. Among various imaging techniques, X-ray radiographs are particularly suitable for applying DL algorithms to medical image analysis. Notably, multi-center research has revealed that radiologists aided by DL-based convolutional neural network (CNN) systems demonstrated an improvement of over 5% in sensitivity (SEN) when identifying malignant lung nodules, compared to radiologists not using such systems [29]. Upon integrating the recommended deep convolutional neural network (DCNN) program, radiologists observed an increase in productivity from 65.10% to 70.30%. Researchers constructed a model based on the ResNet architecture for detecting treatable lung cancer (Figures 5a, 5b). Through a series of transformations, including cropping, scaling, and rotation, they augmented their dataset of 17,211 CXR images to a substantial 600,000. The model exhibited a sensitivity of 76.80% in detecting lung cancer, while the sensitivity of six radiologists reached only 73.20% [30].

Researchers have harnessed deep learning (DL) techniques to automatically classify lung nodules as either normal or pathological [31]. Through the utilization of the Japanese Society of Radiological Technology (JSRT) dataset's 180 segmented chest X-ray (CXR) images (comprising 90 non-nodule and 90 nodule images), a custom deep convolutional neural network (CNN) model was trained and validated. The architecture was enhanced through data augmentation techniques to mitigate overfitting. The model demonstrated a relatively high accuracy (ACC) of 86.67%.

Employing the ResNet-50 model, images from the JSRT dataset were categorized into "no nodule," "benign nodule," or "malignant nodule" categories [32]. The model showcased a sensitivity (SEN) of 92.00% and a specificity (SPE) of 86.00%. Another study employed a substantial dataset of 479,745 CXR scans from a hospital imaging repository [33]. Incorporating attentional feedback into a CNN, the benchmark model achieved a precision (PRE) of 92.00%, a sensitivity (SEN) of 78.00%, an ACC of 85.00%, and an F1-score of 85.00%. In a comparative analysis, a dataset comprising 411 CXR images was employed to train a DCNN RetinaNet model [34]. This model was tested against two radiologists to determine their efficiency in spotting lung nodules in segmented images. Remarkably, RetinaNet's area under the curve (AUC) of 87.00% surpassed that of both human radiologists. Further research explored the utility of DL within the picture archiving and communication system (PACS) at the Third Hospital of Peking University. A dataset of 1,881 chest X-rays (923 diagnosed and 958 normal with pneumoconiosis) was employed for training the Inception-V3 model with fine-tuning. The Inception-V3 model exhibited an AUC of 87.80%, outperforming the AUCs (66.80% and 77.20%) of two human radiologists [35].

**Figure 6:**
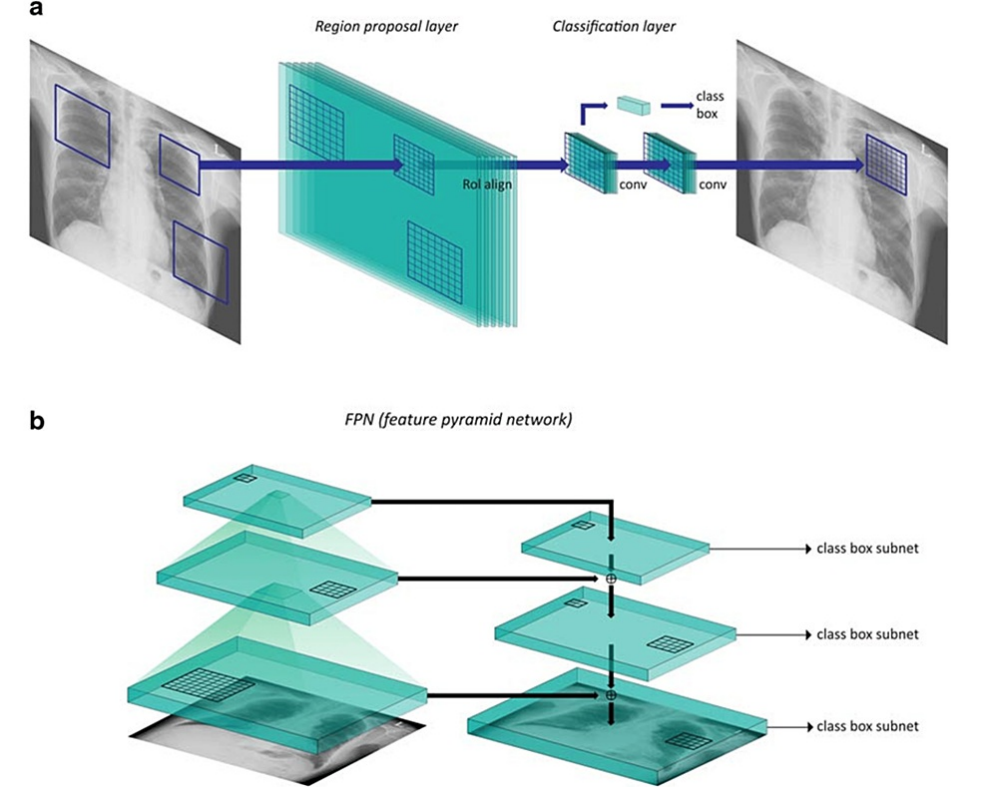
Diagrams of (a) mask R-CNN with region proposal layer and classification layer and (b) RetinaNet with feature pyramid network (FPN). Reproduced with permission from Reference [37]. Copyright 2020 SpringerLink.

Developing a lung nodule detection system based on DenseNet architecture, researchers utilized the JSRT dataset's CXR images after preprocessing steps such as lung region segmentation and bone suppression. The proposed DCNN model significantly outperformed average radiologist predictions, achieving a remarkable accuracy rate of 99.00% [36]. The impact of varying CXR image sizes (256, 448, 896, 1344, and 1792 pixels) was evaluated using two DCNN models (RetinaNet and Mask R-CNN). A dataset consisting of 2,088 CXR images with abnormal results (nodules or masses) and 352 without was employed. Mask R-CNN determined that 1344 pixels provided optimal results, while RetinaNet achieved its best performance with 896 pixels. Both models excelled in the sensitivity (SEN) test, achieving a score of 95.60% (Figure 6) [37].

Tuberculosis detection

The World Health Organization (WHO) has identified tuberculosis (TB) as a prominent contributor to global mortality. Subsequent to the COVID-19 pandemic, TB has overtaken HIV/AIDS as the most significant infectious cause of death on a global scale. In 2020, TB affected approximately 1.1 million children among an estimated total of 10 million cases. In 2019, TB resulted in the deaths of approximately 1.4 million individuals, and this toll is projected to rise to around 1.5 million in 2020. TB, attributed to the *Mycobacterium bacterium*, is an airborne disease that spreads rapidly through respiratory means, particularly when an individual with TB coughs or sneezes. The lungs are frequently affected by this disease [38]. Between the years 2000 and 2020, it's estimated that approximately 66 million lives could have been saved if tuberculosis (TB) had been diagnosed at an earlier stage. Pulmonary tuberculosis can manifest in various ways on chest X-ray (CXR) scans. The successful detection and classification of TB patients using deep learning (DL) techniques have been demonstrated. Researchers have proposed a robust DL network named TBXNet to tackle this challenge and provide a solution for TB detection [39]. TBXNet comprises a total of five two-way convolutional blocks (512, 256, 128, 64, and 32). The architecture's fusion layer combines a pre-existing layer with the two convolution blocks, allowing it to learn new information from the pre-trained layer. An accuracy rate of 99.17% is expected with the envisioned TBXNet. The experiments utilized two labeled datasets: one from the Kaggle public open repository and the other, Montgomery, developed by multiple Belarusian Department of Health institutions.

**Figure 7:**
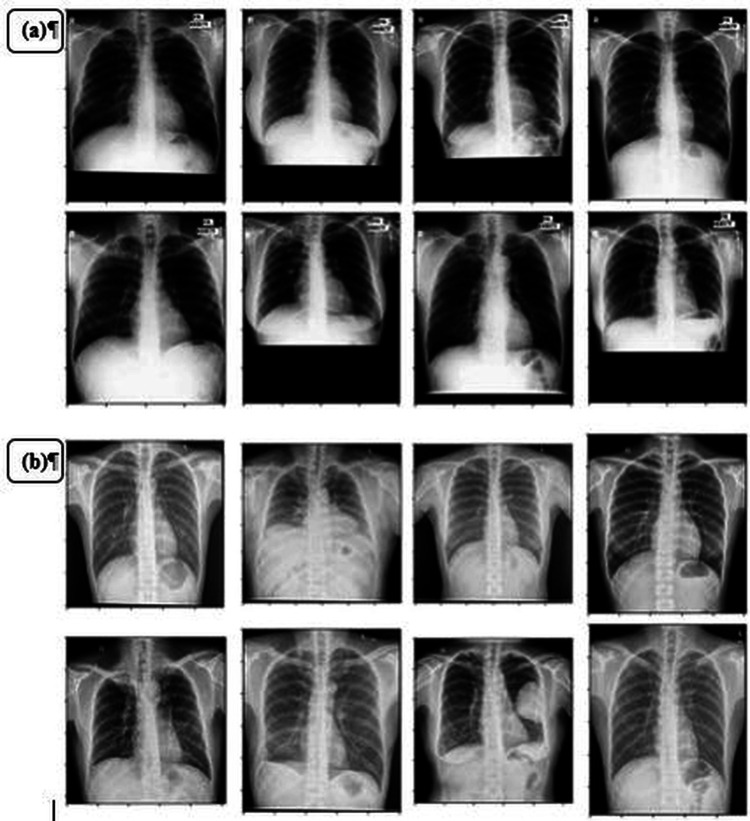
(A) Chest X-ray image samples with labels from the Shenzhen, China dataset and (B) the Montgomery County, Maryland dataset. Reproduce under the terms of the Creative Commons Attribution License from Reference [41]. Copyright 2022 Polish Medical Society of Radiology.

Another suggestion involves automated tuberculosis detection using the VGG16-Coord Attention model [40]. This approach involves introducing a synchronized attention mechanism into the internal structure of the VGG-16 model. This model was one of several deep learning models assessed to determine the most accurate model type for TB diagnosis. The other models considered were VGG-16, MobileNet-V2, Version-Transformer, ResNet-50, and the original Version-Transformer. All models underwent further training for 90 additional epochs after the initial 30 epochs. These experiments were conducted using data from the Shenzhen and Montgomery County, Maryland databases (Figures 7a, 7b). The careful integration of attention mechanisms yielded positive results, evident by a 97.71% area under the curve (AUC), 92.73% predictive accuracy, 92.73% recall accuracy, and 92.82% precision (F1). Furthermore, an entirely novel deep convolutional neural network model for automated TB detection in CXR images has been developed [41].

The researcher employed five pre-trained models (VGG-19, Xception, ResNet-50, VGG-16, and Inception-V3) to assess the transfer learning strategy proposed in the study. Computed tomography images from the Montgomery and Shenzhen databases were utilized for the investigation. Using the recommended DCNN architecture (ConvNet), the PRE, SEN, F1-score, ACC, and AUC achieved a value of 87.0%. For the classification of CXR images from the Shenzhen dataset into TB or normal categories, utilized a dual-convolutional neural network ensemble technique (GoogleNet and AlexNet). Preprocessing strategies such as image contrast enhancement and rotation improved the ACC's performance in cases of uncertain categorization. The employed model yielded an AUC of 99.00%, representing a statistically perfect curve [42]. To surmount the challenges of building a system from scratch and enhance its performance, combined a neural network with a deep convolutional model for AlexNet-like learning using transfer. This approach utilized the Karlsruhe Institute of Technology (KIT), Montgomery, and Shenzhen datasets, achieving an ACC of 90.30 and an AUC of 96.40 [43]. In another study, researchers constructed a DCNN model by merging two CXR datasets [44], collecting tuberculosis and non-TB images from the Chinese city of Shenzhen and the United States' NIH Clinical Centers. Multiple trials demonstrated the high accuracy of the DCNN model (SEN = 72.000%, SPE = 82.000%, and AUC = 98.45%) [44]. Datasets like the Montgomery and Shenzhen ones were chosen due to their public accessibility, and a database comprising 3,500 TB images and 3,500 normal CXR images was compiled. Nine distinct DCNN models were pre-trained and utilized, with DenseNet-201 achieving the highest accuracy (98.60%), precision (98.57%), recall (98.56%), specificity (98.56%), F1-score (98.56%), and recall (98.54%) when segmenting lung images [45].

In a different approach, researchers devised a system employing multiple pre-trained deep convolutional neural network models from the ImageNet dataset. An ensemble technique based on the type-1 Sugeno fuzzy integral was used to determine an average prediction. This method outperformed classification tasks, achieving an ACC of 99.75% using state-of-the-art methods. All the mentioned studies relied on the CXR database [46]. Utilizing the Montgomery and Shenzhen datasets, a study introduced a deep learning method for classifying CXR images as normal or TB. The model employed a convolutional neural network with deep layers, featuring three independent optimizers. Among them, Adam achieved an impressive ACC of 82.09%. Additionally, CXR images were classified as normal or TB using a pre-trained DenseNet system [47]. In another study, the images were sourced from the Shenzhen and Montgomery databases. By merging DenseNet-121 with an enhanced transfer learning technique and the Shenzhen dataset, the model achieved AUCs of 99.00% and 84.00%, respectively [48]. Using an ensemble learning strategy that incorporated various pre-trained deep convolutional neural network models as feature extractors, researchers were able to determine the presence of tuberculosis in CXR images. The evaluation was carried out on accessible datasets from both the Montgomery and Shenzhen databases, with the highest ACC achieved by any model being 80.00%. Moreover, four distinct DCNN models were employed for TB classification, including ResNet-50, VGG-16, GoogleNet, and VGG-19. The research utilized data from public sources in both Montgomery and Shenzhen. The VGG-16 demonstrated superior performance compared to the other three models, with an ACC of 86.74% and an AUC of 92.00% [49]. To address the complexities of data distribution, a class decomposition approach to transfer learning was implemented that enhanced the efficiency of models pre-trained on ImageNet. DCNN models, such as AlexNet, GoogleNet, and ResNet, were employed with and without class decomposition [50]. Another study achieved an ACC of 99.80% on the public JSRT dataset [51].

Detection of COVID-19

In late 2019, COVID-19 emerged in Wuhan, China, and due to its rapid spread and severe impact on humanity, the World Health Organization designated it as a pandemic in early 2020. The detection of COVID-19 in humans often requires a series of costly and time-consuming clinical investigations. Despite this challenge, CXR images have been used to positively identify COVID-19. The death toll from this pandemic has already reached millions, leaving humanity vulnerable to its lingering effects. Deep learning (DL) techniques have been employed to detect and monitor COVID-19-related lung injury through CXR images. Initially, the absence of CXR images for positive cases posed challenges to utilizing DL algorithms for COVID-19 identification and classification during the early stages of the epidemic. To encourage the use of CXR records containing pandemic cases, these records were made publicly accessible, enabling the scientific community to study the virus and devise strategies to curb its spread. Experiments employing various DL approaches and models demonstrated multi-class classification accuracy ranging from 89.01% to 98.00%. For two- and three-class classifications, accuracy reached levels of 90.00% to 99.01%. In a study proposing a deep convolutional neural network model for automated detection of CXR images with COVID-19, a model consisting of five convolutional blocks was introduced [52]. The ReLU activation function was used in each layer of these blocks, and overfitting was mitigated by incorporating a dropout layer between the third and fourth blocks. The model employed a configuration with two fully connected layers (FCLs) and employed dropout and initial fully linked layers. The latter utilized a softmax classifier. This framework achieved a flawless F1-score, a sensitivity (SEN) of 96.00%, and a precision (PRE) of 96.00%. The dataset analyzed comprised 10,293 CXR scans, including 2,874 with COVID-19, 4,200 with pneumonia, and 3,218 categorized as normal. The data was sourced from the COVID Chest repository on Kaggle and an X-ray dataset [53].

**Figure 8:**
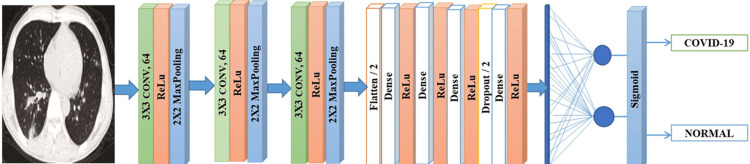
Schematic assembly of the proposed CovMnet model. Reproduce with permission from Reference [55]. Copyright 2022 SpringerLink.

Researchers also developed a DL method for diagnosing COVID-19 using widely available CXR images. The Inception-V4 model was utilized as a trained transfer learner, allowing for automatic detection of COVID-19 in CXR images. This study utilized data from two distinct chest X-ray datasets: Pediatric-CXR (containing images from 1,000 healthy children) and COVID-19 (504 images from patients with the virus). The proposed approach achieved an overall accuracy (ACC) of 99.63% in identifying COVID-19 infection [54]. In the effort to distinguish between typical and COVID-19 CXR images, a DL model named CovMnet was developed, as depicted in Figure 8 [55]. The architecture of CovMnet comprises three layers: ReLU activation, convolution, and MaxPooling. Following the final convolutional layer, the output passes through four dense layers, an activation layer, and a Dropout layer before being flattened and fitted into the neurons of the architecture. Experiments were conducted to assess four variations of the proposed CovMnet model, with the aim of determining the optimal settings. These experiments involved extracting deep features from a dataset and hyperparameter tuning for both convolutional neural networks and full-stack training. The CovMnet model demonstrated an impressive accuracy (ACC) of 97.30%.

The pediatric-CXR dataset was consistently utilized in various CXR image-based research studies. In a study, three methods were employed to identify COVID-19 cases. Two of these methods involved refining models through deep feature extraction and transfer learning, while the third method introduced an entirely new deep convolutional neural network model. For this investigation, the researchers accessed 180 COVID-19 CXR images and 200 normal CXR images from the chest X-ray for chronic obstructive venous disease and pediatric-CXR databases. Each image was annotated by specialists, and data augmentation techniques were utilized in both fine-tuning and full-stack training. Among five pre-trained models optimized for deep feature extraction (ResNet-101, VGG-19, ResNet-50, VGG-16, and ResNet-18), ResNet-50 demonstrated the highest accuracy rates for sensitivity (SEN) at 94.0%, overall accuracy (ACC) at 95.79%, and specificity (SPE) at 97.5% [56]. Another study employed a three-part process to identify pneumonia and COVID. In the first step, CXR image analysis was used to confirm pneumonia. The second step focused on distinguishing between pneumonia and COVID-19 cases. In the final phase, the locations of COVID-19 sightings were pinpointed. This research drew from two extensive chest X-ray datasets, ChestX-ray14 and COVID, encompassing CT and MR images of the chest from various sources. In this context, the VGG-16 model achieved an average accuracy (ACC) of 97.00% [57]. A DL model for COVID-19 patient recognition was trained using a CT scan and 400 CXR images, including 500 typical COVID-19 cases. The hyperparameters of eight distinct DL models were fine-tuned. Among them, NasNetMobile demonstrated the highest accuracy (93.94%) among the tested models [58].

Another research effort aimed to differentiate COVID-19 from pneumonia using a deep convolutional neural network model [59]. Researchers examined various image collections, including data from COVID-19 and the Pediatric-CXR pediatric X-ray dataset available on Kaggle [60]. Five models (Inception, VGG-19, MobileNet-V4, Inception, and ResNet-v2) were trained using transfer learning parameters. MobileNet-V2 exhibited superior performance with an accuracy of 96.78%, sensitivity of 98.66%, and precision of 96.46%. Lastly, in a study involving a DenseNet-121 DCNN model, 21,165 images from datasets such as Radiology Collaborated to Develop the RSNA International COVID-19 Open Radiology Database (RICORD), radiography for COVID-19, CXR in children, and BIMCV-COVID-19+ were used for training [61]. Applying the proposed model to the binary classification of COVID-19 cases using HE and geometric data augmentation approaches resulted in an ACC of 97%. A deep convolutional neural network model was suggested for pandemic recognition [62]. The training dataset encompassed nine types of pneumonia depicted in photos sourced from the COVID-19 chest X-ray public dataset, which includes 316 CXR images [63]. To counter overfitting, data augmentation techniques were employed. The suggested model attained an ACC of 96.00% [64]. In a study, the performance of 13 distinct DCNN models (Shuffle Net, Inception, ResNet-V2, AlexNet, Resnet-18, Resnet-50, Inception-V3, Densenet-201, MobileNet-V2, VGG-19, VGG-16, Resnet-101, Xception, and GoogleNet) was examined. Out of these models, VGG-19 and COVIDx CXR-3 produced the highest ACC (99.81%) when tested on a dataset containing 700 images, with 350 of them being COVID-19 cases and 350 normal cases [65]. For disease classification, a rational binary and multi-disease grouping approach was proposed. An optimal DCNN model, EfficientNet-B5, was trained on nine datasets, and the pediatric-CXR dataset combined with the COVIDx repository contributed over 3200 CXR images to the research [66]. The utilization of gradient-weighted class activation mapping (Grad-CAM) helped simplify the interpretation of heatmaps. EfficientNet-B5 achieved an AUC of 98.00% for binary classification of COVID-19 cases and 97.00% for multi-classification involving pneumonia, COVID-19, and normal cases [67]. A method for automated COVID-19 identification, employing COVIDX-Net technology, was proposed. The framework supported seven unique DCNN designs (DenseNet-201, Xception, InceptionResNet-V2, VGG-19, Inception-V3, ResNet-2, and MobileNet-V2) and was trained using the COVID chest X-ray dataset. F1 scores of 89.00% and 91.00% were achieved by the VGG-19 and DenseNet models for normal and COVID-19 cases, respectively [68]. A study employed data augmentation and classification for CXR images, categorizing them into four classes: lung opacity, pneumonia, COVID-19, and normal. EfficientNet-B1 outperformed its counterparts with an ACC of 96.13% [69]. A study included a total of 21,165 CXR images from datasets including radiography for BIMCV-COVID19+, RSNA pneumonia CXR, COVID-19, and pediatric CXR. This involved a custom ResNet model utilizing multi-head self-attention for categorizing CXR images into pneumonia, COVID-19, and normal categories. The study employed a gray-level co-occurrence matrix (GLCM) technique to extract textured details from CXR images. The analysis utilized COVIDx CXR-3 software and a dataset consisting of 5173 individual CXR images, achieving an ACC of 95.52 and a PRE of 96.02 [70].

Multiple disease detection

It is possible for a patient to concurrently experience multiple diseases, significantly elevating the risks to their life. Given the overlapping symptoms of various illnesses, diagnosing multiple pathologies through CXR images can be a challenge for radiologists. In such cases, further research and testing might be necessary. Several deep learning (DL) systems have been devised, employing diverse strategies to address this complexity. In an effort to differentiate between pulmonary nodules and cardiomegaly disorders in CXR images, a study introduced a customized DenseNet-121 model. They utilized images from the CheXpert dataset for this study. The model demonstrated an AUC of 73,000% for detecting lung nodules and 92,000% for detecting cardiomegaly [71]. A DL approach was employed that merged data extracted by a deep convolutional neural network model with low-level characteristics to identify cardiomegaly, normal/abnormal conditions, and pleural effusion. Their dataset comprised 193 chest X-rays from Sheba Medical Center, preprocessed using the described methods. The results indicated an AUC of 93,000% for pleural effusion, 89,000% for cardiomegaly, and 9,000% for normal/abnormal classification [72].

In the realm of identifying abnormalities in chest radiographs, the GoogleNet model was used to classify chest images based on the presence of abnormalities such as consolidation, normal tissue, cardiomegaly, pneumothorax, pleural effusion, and pulmonary edema. AUC scores for GoogleNet's models were 86.80%, 96.20%, 86.10%, 96.40%, and 96.40% for edema, pleural effusion, pneumothorax, normal, cardiomegaly, and consolidation, respectively [73]. This research illustrates the successful training of DCNN models on limited medical datasets without compromising performance. A study utilized a weak-supervised approach for classifying and detecting eight chest disorders from the ChestX-ray8 dataset, achieving an AUC of 80.30% for detecting significant anomalies [74]. Using the ChestX-ray8 dataset, a DenseNet model was employed to extract disease characteristics and demonstrated a relatively high area under the curve (AUC) of 79.80% [75]. Employing the DenseNet-121 model and data from ChestX-ray14, a study achieved state-of-the-art results with an average AUC of 84.11% for classifying the 14 diseases in the dataset [21]. To classify CXR images into normal, lung disease, and heart illness categories, an ensemble learning strategy was proposed by combining photos from the VinDr-CXR and CheXpert databases. This strategy, along with data augmentation, achieved an average AUC of 94.89% [76]. A cascading neural network was used to classify the 14 illnesses from the ChestX-ray14 dataset. The presented model achieved performance levels comparable to state-of-the-art methods, with an average AUC of 79.50% [77]. To identify 14 chest diseases from CXR images, an AMDenseNet model with an attention mechanism was suggested. Their DenseNet-121-based models outperformed prior research in terms of average AUC [77,78].

A comprehensive approach utilizing transfer learning was used to categorize CXR images into normal, pneumonia, and pneumothorax categories. EfficientNet-V2M achieved impressive results with an average ACC of 82.15%, SEN of 81.40%, and SPE of 91.65% [79]. Similarly, an average ACC of 82.20% was obtained across four classes (normal, TB, pneumonia, and pneumothorax) using a model on a dataset from Cheonan Soonchunhyang University Hospital [80]. Another study employed multiple models based on binary relevance for identifying chest diseases in the CheXpert dataset. The Xception DCNN model, in combination with the Adam optimizer, achieved superior results, yielding an overall mean AUC of 94.90% across all disorders [81]. For a summary of CXR-based detection of various chest diseases and the employed models, Table 1.

**Table 1:** CXR-based detection of various diseases. CXR: chest X-ray, ACC: accuracy, SEN: sensitivity, SPE: specificity, PRE: precision, MHSA: multihead self-attention network, BRISK: binary robust invariant scalable key-points, RICORD: Radiology Collaborated to Develop the RSNA International COVID-19 Open Radiology Database, RSNA: Radiological Society of North America, JSRT: Japanese Society of Radiological Technology.

S. no	Dataset	Results	Diseases	Model	References
1	Pediatric-CXRs	ACC = 97.20%, ACC = 87.30%	Pneumonia	Full-layer connected Swin transformer	[18]
2	Using CheXpert, we gathered a total of 2440 images: 2088 with nodule and 352 normal.	SEN = 95.60%	Lung nodule	Overlay RetinaNet with R-CNN.	[37]
3	Extracted from the pediatric-CXR dataset, 648 CXR pictures	ACC = 97.40%	COVID-19	Our own personal convolutional neural network (CovMnet)	[55]
4	JSRT	SEN = 98.00%, ACC = 99.80%, SPE = 99.00%	Tuberculosis, tumor	Class decomposition-based deep convolutional neural network model (ResNet)	[50]
5	RSNA-pneumonia-CXR	ACC = 94.00%	Pneumonia	Insight-V4 using learned transfer	[20]
6	Peking University Third Hospital PACS had 1881 chest x-rays (958 normal, 923 pneumoconiosis).	AUC = 87.80%	Pneumoconiosis	Improved inception-V3	[35]
7	Montgomery and Shenzhen	PRE = 88.00%, AUC = 87.00%, SEN = 87.00%	Tuberculosis	A freshly trained ConvNet model	[41]
8	400 Pediatric-CXRs	ACC = 93.94%	COVID-19	NasNetMobile	[58]
9	X-viral (5977 viral pneumonia and 37,393 images of pneumonia that are not caused by viruses) and X-COVID-19 (106 COVID-19, 107 normal) datasets.	AUC = 83.61% SEN = 71.70%	COVID-19	Anomaly detection model with confidence	[82]
10	17,211 CXRs for training (600,000 pictures) tests, and 10,285 people (1,483 diagnosed with lung cancer).	AUC = 73.20%, SEN = 76.80%	Lung cancer	Two different ResNet models, ResNet-50 and ResNet-101	[30]
11	Pictures from Montgomery, a Kaggle repository and a Belarusian Ministry of Health dataset	ACC = 99.17%	Tuberculosis	A specialized TBXNet DCNN model	[39]
12	Radiography for pandemic, BRISK, RICORD for COVID-19+ and CXR in children	ACC = 97.00%	COVID-19	DenseNet-121	[61]
13	Pediatric-CXR	ACC = 83.38%	Pneumonia	A CNN model with and without extra data is compared	[23]
14	JSRT segmented CXR pictures (90 non-nodule and nodule images).	AUC = 86.67%	Lung nodule	Built-to-order deep convolutional neural network with extra data	[31]
15	Shenzhen and Indiana	SEN = 72.00%, SPE = 82.00%, AUC = 98.45%	Tuberculosis	The InceptionV3 learning transfer module	[44]
16	CXR-3 COVIDx	The F1 score is 91.00%	COVID-19	VGG-19	[68]
17	Custom dataset containing 5173 COVIDx CXR-3 pictures.	ACC = 95.52%, PRE = 96.02%	COVID-19	A specialized deep convolutional neural network (MHSA-ResNet)	[70]

Advancements to MRI

MRI offers several advantages compared to standard CT and PET/CT scans, including the ability to avoid the use of a radioactive contrast agent during PET/CT scans [83]. The diagnostic utility of MRI in lung cancer diagnosis has grown thanks to advancements in MRI techniques. One notable fast MRI sequence is known as turbo-spin echo (TSE), which has the potential to detect malignant nodules at a rate comparable to multidetector computed tomography (MDCT) [84]. TSE is resilient to environmental factors like air and lung tissue susceptibility. The raw magnetic resonance data (k-space) was processed using the "mirror-image" characteristics of the half-Fourier single-shot TSE sequence, resulting in significant improvements in scan speeds [85]. To enhance soft-tissue visualization and local tumor progression detection, TSE-assisted short-tau inversion recovery sequences have been utilized to attenuate lipid-related signals. These sequences can enhance the contrast of pulmonary lesions without requiring prolonged breath-holding periods. However, instances of substantial blood flow over the lungs can still lead to flow artifacts in these sequences, particularly when cardiac gating is insufficient, even when a second recovery sequence is employed [86]. In addition to TSE-based sequences, other studies have found value in radio-frequency spoiled 3D gradient recall echo sequences such as volumetric interpolated breath-hold examinations. This type of sequence has shown reduced motion artifacts, although it might miss very small lesions [87].

The lung parenchyma's short T2* duration (1-2 ms at 1.5 T) presents challenges in MRI imaging. To overcome this limitation, specific technical capabilities are required, such as ultra-short echo (UTE) times and balanced steady-state free precession (bSSFP). Ultra-short echo (UTE) techniques employ very low echo times and k-space radial sampling to enhance signals from tissues with exceptionally short T2/T2* relaxation times, like cortical bone and lung parenchyma. Balanced steady-state free precession employs "balanced" gradients and very short repetition times (TR) to maintain a nearly constant "steady-state" signal by preventing magnetization from relaxing and dephasing throughout a TR repeat [88]. In relation to making definitive malignancy diagnoses with MRI, a literature review on magnetic resonance data, bSSFP, and UTE was conducted. The authors explored potential roles for MRI in the future, ranging from having no role at all to playing a role depending on future assessments of its diagnostic efficacy and cost-effectiveness, potentially alongside or instead of CT. While they found the technology promising, they couldn't definitively conclude that MRI led to improved patient outcomes [89]. Notwithstanding these limitations, the superior soft tissue contrast and ability to detect local invasion of surrounding tissues make MRI the preferred imaging modality for staging lesions near the mediastinum, vertebral body, and chest wall [90,91]. Innovative sequencing and encoding techniques have the potential to enable the capture of images during free breathing, eliminating the quality loss caused by motion artifacts. While radial and spiral acquisitions can effectively reduce ghosting and motion-related artifacts, they are less efficient compared to standard Cartesian k-space acquisition, as they extend the acquisition time and introduce streak artifacts. A study on compliance and noncompliance demonstrated the feasibility of performing free breathing and intermediate anatomical evaluations [91]. In real-world performance comparisons, StarVIBE outperformed the gold-standard dynamic contrast-enhanced (DCE) MRI [91]. Whole-body MRI has historically been limited in its ability to effectively detect potential metastatic diseases due to significant motion-related challenges. However, a comprehensive assessment of its utility extends beyond the scope of this study [92]. Nevertheless, recent findings suggest that advancements in technology have made whole-body MRI a more viable option for early staging [93].

Lung functional imaging

The lung has become a focal point for an increasing number of functional MRI sequences, originally developed for studying other diseases. One such method is diffusion-weighted imaging (DWI), which can differentiate hypercellular regions (tumors) from areas with increased diffusivity by detecting signal attenuation resulting from the restricted diffusion of water. The apparent diffusion coefficient is a measure used in DWI to quantify diffusion [94]. In the complex clinical context of lung cancer with atelectatic lung, DWI surpasses CT and PET/CT [95]. Unlike CT, DWI has the potential to identify lymph node involvement, intratumor vasculature, and effusions. However, DWI might exhibit geometric distortions due to variations in lung susceptibility between tissue and air interfaces, necessitating more advanced technical solutions [96]. Dynamic contrast-enhanced magnetic resonance imaging (DCE-MRI) offers functional insight into blood flow and vascular permeability, shedding light on tumor vascularity patterns [96]. While successfully applied to disease sites like prostate tumor staging [97] and primary brain tumors [97-99]. DCE-MRI initially faced challenges in lung imaging due to breathing-induced image degradation [100]. Presently, the transfer constant from DCE-MRI and the apparent diffusion coefficient from the intravoxel incoherent motion DWI model are employed in clinical practice to distinguish between lung cancers and isolated pulmonary nodules [101]. When comparing DCE-MRI to PET-CT for lung cancer, the standardized uptake value is related to the standard deviation of the middle peak [102]. Both DWI and DCE-MRI may suffer from poor spatial resolution, especially in the presence of breathing and heart rate variability. However, these issues can be mitigated through respiratory/cardiac gating and faster temporal imaging [96,100].

Nuclear medicine imaging

Traditional nuclear medicine imaging often relies on planar imaging techniques, but the utilization of continuous (cine) imaging can have a significant impact on indications such as lymphatic flow assessment. In conventional gamma cameras, one or more NaI(Tl) crystals are employed to detect gamma rays emitted from the radio-tracer inside the patient's body. During a single photon emission computed tomography (SPECT) scan, the patient is surrounded to capture multiple two-dimensional images (projections). Subsequently, a tomographic reconstruction technique is applied to these projections to create a spatial model. The combination of SPECT with planar imaging can enhance diagnostic accuracy and disease severity assessment [103,104]. However, SPECT alone may not provide precise localization. To address this limitation, a hybrid imaging modality called single photon emission computed tomography/computed tomography (SPECT/CT) has been introduced [105]. In SPECT/CT, CT is used for attenuation correction and precise anatomical localization. This combination enhances the reliability of imaging results compared to planar imaging [106]. SPECT/CT has demonstrated superiority over planar scintigraphy or standalone SPECT [107,108] in various established indications and emerging applications. To minimize radiation exposure, CT can be used in a low-dose mode for diagnostic purposes. The effective radiation dose from the CT component of a SPECT/CT examination can vary from 0.6 mSv to 2.6 mSv, depending on the scanned body area [109,110]. For context, the average annual background radiation exposure in the United States is around 3.1 mSv, and a typical chest CT scan delivers about 7 mSv of radiation dose [111].

**Figure 9:**
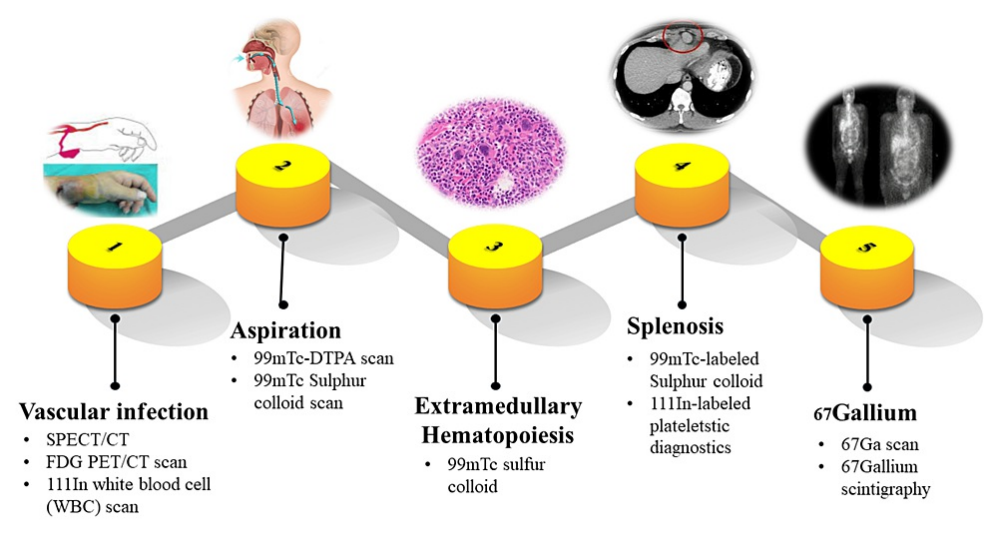
Nuclear medicine used for the detection of various diseases. SPECT: single photon emission computed tomography, CT: computed tomography, PET: positron emission tomography.

Molecular imaging acts as a bridge between anatomical and molecular data by combining various imaging techniques such as SPECT, PET, MRI, ultrasound, and optical imaging, along with specific imaging probes (as shown in Table 2). Tagged probes with molecular specificity enable the exploration of specific aspects of cellular pathology [112]. Currently, molecular imaging using SPECT and PET is in use in clinical practice. While SPECT has lower resolution compared to PET, it has the capability to simultaneously scan multiple molecular probes using different radiotracers. Ongoing research is focusing on developing new PET techniques that can image multiple radio-pharmaceuticals simultaneously, although further advancements are necessary [113]. Recent pre-clinical studies have explored alternative techniques, including optical imaging with fluorescent probes and contrast-enhanced molecular ultrasound. Molecular imaging has the potential to reveal subtle information that could lead to more precisely targeted therapies. Figure 9 illustrates the diverse applications of nuclear medicine in detecting various diseases.

**Table 2:** Combination of nuclear medicine and other techniques. FDG: fluorodeoxyglucose, FUO: fever of unknown origin, VQ: ventilation-perfusion.

S. no	Modality	Radiotracers	Advantages	Description	Indications/examples	References
1	PET (positron emission tomography PET/CT)	^18^FDG—nonspecific multiple newer tracers	Higher resolution than SPECT. Newer tracer-targeted molecular imaging. Metabolism evaluation (FDG).	Usually with CT for lesion localization and attenuation correction.	Oncologic imaging. Infection/inflammation (sarcoidosis, FUO, cardiac).	[114]
2	Gamma camera	Gamma-emitters (81Tl, 131I, 123I, ^99^mTc, 111In, etc.).	Unlike CT and radiography, functional imaging provides functional data.	Planar imaging, flow/cine.	The gold standard of nuclear medicine, radiology covers a wide range of medical conditions.	[115]
3	MRI (PET/MR)	Tracer PET superparamagnetic iron oxide (USPIO) particles are very tiny in size.		Magnetic resonance imaging used in conjunction with PET	Imaging for cancer, particularly for common forms including prostate cancer, cervix, and liver cancer.	[116]
4	Single-photon emission computed tomography (also known as SPECT or SPECT/CT)	Planar imaging is easily augmented with gamma-emitting radionuclides as required.	Compared to planar imaging, this method of lesion localization is more accurate and precise.	Formats in many planes are feasible. A tomographic reconstruction method yields three-dimensional information.	Localization of the parathyroid glands, myocardial perfusion imaging, and a VQ scan.	[117]
5	Optical Imaging	Fluorophores and quantum dots. Luciferase enzyme.	Limited depth	Fluorescence, bioluminescence	Pre-clinical	[118]

Vascular infections

Patients who fall under the category of "possible infectious endocarditis" according to the Duke criteria, particularly when there is significant echocardiographic data that contradicts clinical suspicion, are the most suitable candidates for a labeled leukocyte scan aimed at identifying infectious endocarditis [119,120]. Radiolabeled white blood cell SPECT/CT is more effective in identifying infectious foci and implanted prosthetic valve infections compared to FDG PET/CT [121]. FDG PET/CT, on the other hand, is valuable in detecting septic emboli and determining the source of infection outside the heart [122]. For cases where mycotic aneurysms are not easily distinguishable through CT or MR imaging, a 111In white blood cell (WBC) or FDG PET/CT scan can aid in diagnosis and locating other disease sites in individuals with such abnormalities [123]. Labeled white blood cell imaging demonstrates high sensitivity when applied to vascular grafts and can be used to detect, locate, and quantify infection. However, early postoperative evaluation remains a challenge [124,125]. A positive result on a postoperative scan might indicate either infection or healthy graft endothelialization. White blood cell scintigraphy, especially when combined with SPECT/CT technology, is a tool that can assess the presence of infection in cardiac implantable electronic devices (CIED) [126]. Despite evidence suggesting that WBC SPECT/CT and FDG PET/CT scans could aid in diagnosing CIED infection, these tests were not recommended by the European Society of Cardiology in their 2015 recommendations [127,128].

Aspiration

The diagnostic aids for a salivary ^99^mTc sulfur colloid scan with ^99^mTc-diethylenetriamine pentaacetate (DTPA) demonstrate higher sensitivity compared to fluoroscopic procedures. It's noteworthy that video fluoroscopic swallowing assessment can fail to detect aspiration in around 30% of patients, especially in instances involving saliva aspiration [129,130].

Extramedullary hematopoiesis

The differentiation of this condition from other paraspinal lesions can be achieved through the utilization of ^99^mTc sulfur colloid. Pulmonary extramedullary hematopoiesis encompasses the respiratory system, including the lungs, chest cavity, and occasionally the pulmonary artery. Hence, this approach could potentially offer advantages in managing such cases [131].

Splenosis

Scintigraphic imaging is a common approach when there are indications of thoracic splenosis, a condition that can arise following a splenic injury. Among individuals with splenosis, 75% exhibit multiple nodules on their pleura, whereas the remaining 25% present just one nodule. Notably, patients often endure a wait of more than 21 years before receiving a diagnosis [132]. Scintigraphy can facilitate diagnosis using various techniques, such as ^99^mTc-labeled sulfur colloid, ^99^mTc-labeled heat-damaged erythrocytes, or 111In-labeled platelet diagnostics (theranostics).

^67^Gallium

The current pneumonic plague, attributed to *Pneumocystis jirovecii*, and nearly all other lung-related ailments can be ruled out with a negative scan. In cases of immunocompromised patients, ^67^Gallium scintigraphy becomes a valuable tool for identifying opportunistic infections in both the lungs and mediastinum. Conditions marked by granulomatous inflammation, like sarcoidosis and tuberculosis, can be diagnosed or monitored using ^67^Ga [133,134]. The test is effective in detecting toxic effects on the lungs caused by drugs [135]; it is particularly preferred for leukopenic patients over white blood cell scans. Notably, compared to labeled leukocytes, ^67^Ga provides a more precise diagnosis for disc and osteomyelitis. Furthermore, a ^67^Ga scan aids in the diagnosis of conditions such as adenocarcinoma, Kaposi sarcoma, and histiocytosis lymphoma, unlike squamous cell carcinoma. Nonspecific pulmonary absorption of ^67^Ga is associated with various viral and inflammatory disorders. However, due to lengthy testing times, significant potential for errors, substantial radiation exposure, and the availability of alternative options, the importance of ^67^Ga scintigraphy in clinical practice has waned [110,136].

Labeled leukocytes in pulmonary infections

Depending on the specific clinical scenario and desired leukocyte localization, the utilization of 111In-Oxone (also known as ^99^mTc-HMPAOxime) can effectively label a patient's white blood cells. White blood cell (WBC) imaging serves as a valuable tool in identifying patients with fever, positive blood cultures, and granulocytosis, as recommended by the Society of Nuclear Medicine and Molecular Imaging, to infer acute inflammation or infection at specific sites [137,138]. According to recommendations from the European Association of Nuclear Medicine, labeled white blood cell scintigraphy can be employed for diagnosing occult lung infections, postoperative abscesses, endocarditis, infections associated with vascular devices, infected central venous catheters, and determining the severity of these conditions [139]. However, there are certain limitations associated with scintigraphy using radiolabeled WBCs. These include the necessity for manual blood handling during radiopharmaceutical preparation, longer procedure times in comparison to techniques with lesser spatial resolution like 18F-2-deoxyglucose PET/CT, and the potential for inconclusive results due to sequestration of injured leukocytes in the lungs. In cases of diffuse uptake, particularly in patients with cardiac or renal failure, lung infection or inflammation could be the underlying cause, potentially obscuring specific areas of lung disease [140]. Diffuse uptake patterns might indicate conditions such as sepsis, septic shock, or atypical lung infections like *Pneumocystis jirovecii*. Similar clinical presentations are seen in acute respiratory distress syndrome (ARDS), eosinophilic syndromes, graft-versus-host disease, and lung damage induced by drugs or radiation. Focal uptake on delayed images might be lobar/segmental or non-anatomical in distribution. Segmental or lobar uptake suggests pneumonia, while non-anatomical uptake areas indicate technical errors [137,141]. Although ^99^mTc-HMPAO and 111In-oxine exhibit high specificity for neutrophils, their affinity for eosinophils can lead to false positive results in conditions characterized by eosinophilic infiltration [142]. For assessing the efficacy of chronic obstructive pulmonary disease treatment, lung neutrophil inflammation can be quantified using labeled neutrophils [143].

Pneumonia computed tomography-scan

The significance of CT scans in pneumonia diagnosis has garnered increased attention, particularly in emergency departments. A study noticed that out of 319 patients who arrived at the emergency room suspected of having community-acquired pneumonia (CAP), 100 of them experienced an improved prognosis due to early CT scans. In 80% of cases (25% of the total), the final classification by the adjudication committee aligned with the updated CAP probability, factoring in all collected information, including follow-up data. This resulted in a net improvement of reclassification accuracy by 60 out of 319 cases (19%). In most instances, the appropriate correction involved decreasing the likelihood of a pneumonia diagnosis [135]. Another study examined 200 elderly patients, of which 54 individuals (27%) had their pneumonia risk influenced by a low-dose CT scan. In 65% of these cases (17.5%), an adjudication committee, blinded to the low-dose computed tomography (LDCT) scan findings, deemed the changes in pneumonia likelihood level as appropriate. Among 200 patients, only 16 experienced a positive change in their overall classification. Notably, patients initially misclassified as having pneumonia but later reclassified as not having the condition showed the highest success rate. CT scans have the potential to reduce the occurrence of incorrect pneumonia diagnoses [137]. These studies also emphasize the utility of CT scanning in both clinical and emergency care settings. While conventional chest radiographs typically expose patients to radiation levels of 0.05-0.03 mSv, with average annual background radiation levels around 4 mSv, low-dose CT scans can be completed in as little as 10 minutes. Furthermore, nearly a third of patients showed additional radiological abnormalities, with lung nodules occurring in about 10% of cases. While these findings can aid in identifying and treating previously undetected diseases, they also pose the risk of being excessive, particularly for the older population.

## Conclusions

The article critically assessed deep learning CAD techniques for chest-based illness diagnosis utilizing MRI, CXR, nuclear medicine, and CT scans. CADe/CADx systems and deep learning developments were briefly covered. Chest sickness was found by comparing MRI, CXR, nuclear medicine, and CT. Our extensive analysis demonstrates that deep learning has been used in pulmonary nodule technology many times. This place is hard. The difficulties were thoroughly addressed, and research options were explored. Interpretability, overfitting, and poorly annotated datasets are concerns. CAD research centers using deep learning. Deep learning and understanding research is vital. A CAD system that describes outcomes helps radiologists diagnose. Models for multi-modal tumor detection, classification, segmentation, and more will be created using deep learning algorithms and study photos. This method provides complete diagnostic information for patient assessment and treatment. Data analytics-driven AI like machine and deep learning will help radiology. Data analytics-driven learning may speed radiology imaging scan chest diagnosis, research finds. Early chest-based illness detection improves treatment, prevention, and mortality. Obtaining fresh data is tough, but a growing dataset that allows thorough diagnosis and recognizes diagnostic limits is crucial. All diagnosis elements must be covered by a high-quality dataset with distinct properties for unified detection, classification, and segmentation.
